# Cultural Individualism–Collectivism and Third‐Party Punishment and Compensation

**DOI:** 10.1002/pchj.70061

**Published:** 2025-10-22

**Authors:** Yan Ye, Zuo‐Jun Wang

**Affiliations:** ^1^ School of Public Administration Hohai University Nanjing People's Republic of China; ^2^ School of Educational Science Anhui Normal University Wuhu People's Republic of China

**Keywords:** cultural individualism–collectivism, third‐party compensation, third‐party punishment

## Abstract

This study examined how culture shapes third‐party punishment and compensation in the harm domain using realistic judicial scenarios. Chinese participants showed greater engagement in both forms than American participants, with individualism–collectivism values mediating these societal differences.

## 1 Introduction

When others are harmed or treated unfairly, observers may incur personal costs to punish violators or compensate victims—a phenomenon known as third‐party intervention. This mechanism plays a crucial role in maintaining social cooperation (Guo et al. 2024). Research shows that third‐party intervention is not only shaped by individual factors, such as cognitive development, but also by sociocultural contexts (Wang et al. 2025). However, prior work has mainly focused on punishment, neglecting alternative responses such as compensation. It has also centered on distributive fairness while overlooking violations of harm norms—a core and cross‐culturally universal moral principle (Schein and Gray 2017). Finally, cross‐cultural studies often use country as a crude proxy for cultural values, leaving the nuanced role of culture unclear, given other group‐level differences (e.g., economic development, political institutions).

To address these gaps, we directly compared punishment and compensation as forms of third‐party intervention among participants from a prototypical individualistic culture and a prototypical collectivist culture. By incorporating validated measures of cultural values, we offer a more fine‐grained account of how cultural orientations shape these behaviors. Examining responses to harm norm violations through ecologically valid judicial vignettes also informs theories of moral universality and has practical implications for international judicial contexts.

Individualism–collectivism is a core cultural dimension (Hofstede 2001), with the United States and China commonly regarded as prototypical representatives (Feinberg et al. 2019). We therefore compared participants from these two countries. In collectivist contexts, where interdependence is emphasized, severe transgressions are perceived as threats to social order (Sullivan et al. 2016). Consequently, collectivists may impose harsher punishments to deter violations and restore cohesion. At the same time, their relational orientation may promote greater compensation to support victims and repair social bonds (Wu et al. 2025). Thus, compared with those from individualistic cultures, collectivists are expected to both punish more severely and compensate more generously when harm‐related norms are violated.

This study tested these hypotheses. Because harm severity may moderate cultural effects on third‐party intervention (Feinberg et al. 2019), we also manipulated harm severity on an exploratory basis to assess whether the predicted cultural patterns were consistent across different levels of transgression.

## 2 Method

A total of 110 Chinese participants (six failed attention checks and were excluded; 74 female; *M*_age_ = 33.45, SD_age_ = 7.39) from Credamo and 100 American participants (46 female; *M*_age_ = 41.78, SD_age_ = 12.67) from Prolific were recruited, meeting a priori power requirements. After providing informed consent, participants first completed a demographic questionnaire, followed by the individualism–collectivism subscale of the Cultural Values Scale (Yoo et al. 2011; Chinese version obtained through translation and back‐translation). This subscale includes six items (e.g., “Individuals should sacrifice self‐interest for the group,” rated from 1 = *Strongly Disagree* to 5 = *Strongly Agree*) and treats individualism–collectivism as a continuum, with higher scores reflecting stronger collectivist orientations (American: *α* = 0.85; China: *α* = 0.89). Participants then completed the 10‐rung MacArthur Scale of Subjective SES (see Supporting Information S1: A).

Participants from China and the United States were randomly assigned to either a mild or severe harm condition in a between‐subjects design, using four criminal vignettes: traffic crime, poisoning, physical assault, and robbery (see Supporting Information S1: B). For each vignette, they indicated on two separate measures how much of a hypothetical 100‐unit daily income they would donate to a crowdfunding effort to hire a lawyer, aimed at punishing the perpetrator and compensating the victim, respectively. It is important to note that donations for punishment and compensation were not mutually exclusive (see Supporting Information S1: C). Finally, they rated the victim's harm severity on an 11‐point scale (0 = *Not severe*, 10 = *Extremely severe*) as a manipulation check.

All analyses were conducted in R (v4.4.3). Composite scores for punishment and compensation were created by averaging responses across vignettes (see Supporting Information S2: A) and Box‐Cox transformed to better meet the normality assumption of residuals (See Supporting Information S2: B). These transformed scores were entered into ANCOVAs with country, severity, and their interaction as fixed factors, controlling for gender, age, and SES. Mediation analyses (5000 bootstraps) further tested the indirect effect of country via individualism–collectivism scores, with the same covariates included.

## 3 Results

A manipulation check confirmed that participants perceived severe outcomes as more harmful (*M* = 8.36, SD = 1.09) than mild ones (*M* = 5.07, SD = 2.46), *t*(202) = 12.52, *p* < 0.001, *d* = 1.75 (See Supporting Information S2: C).

*ANCOVA Analyses*: For punishment, both the interaction between country and severity, *F*(1, 197) = 0.04, *p* = 0.838, and the main effect of severity, *F*(1, 197) = 0.53, *p* = 0.466, were nonsignificant. Yet, a significant main effect of country emerged, with Chinese participants assigning more punishment (*M* = 8.84, SD = 3.15) than Americans (*M* = 5.07, SD = 3.48), *F*(1, 197) = 25.21, *p* < 0.001. For compensation, both the interaction, *F*(1, 197) = 0.02, *p* = 0.896, and the main effect of severity, *F*(1, 197) = 1.73, *p* = 0.190, were nonsignificant. Yet, a significant main effect of country emerged, with Chinese participants assigning more compensation (*M* = 8.78, SD = 3.55) than Americans (*M* = 6.41, SD = 3.95), *F*(1, 197) = 7.79, *p* = 0.006.

*Mediation Analyses*: Chinese participants reported higher individualism–collectivism scores (*M* = 3.87, SD = 0.77) than US participants (*M* = 3.29, SD = 0.86), *t*(202) = 5.05, *p* < 0.001, *d* = 0.71, confirming country differences. Individualism–collectivism significantly mediated the effect of country on both punishment (*b* = −0.91, *p* < 0.001, 95% CI = [−1.50, −0.45]) and compensation (*b* = −1.06, *p* < 0.001, 95% CI = [−1.71, −0.54]) (see Supporting Information S3).

## 4 Discussion

Our findings show that collectivism promotes both third‐party punishment and compensation. The punishment effect aligns with prior work (Feinberg et al. 2019), while the compensation effect addresses a key literature gap. To the best of our knowledge, this is the first cross‐country comparison on third‐party compensation. Moreover, mediation analyses confirmed that cultural values—rather than country alone—underlie this dual third‐party intervention tendency.

Although we did not directly measure the underlying motives for the two forms of third‐party intervention, our findings are consistent with theoretical perspectives suggesting that collectivist cultures promote social harmony (Sullivan et al. 2016) and place greater emphasis on attending to and empathizing with others' misfortunes (Wu et al. 2025). From this perspective, third‐party punishment and compensation may function as complementary mechanisms for restoring collective moral order. The absence of a moderating effect of harm severity further suggests that our judicial vignettes elicited a strong and consistent justice motive across conditions—one powerful enough to override variations in the perceived severity of the transgression.

This study has several limitations. First, we only compared participants from China and the United States; future research could include additional countries and cultures to test the generalizability of these findings. Second, we focused on the harm norm, and future studies should examine whether the results extend to other moral domains, such as distributive fairness. Third, our study primarily assessed third‐party intervention intentions, and future research could investigate actual third‐party behaviors more directly.

Despite these limitations, our findings theoretically demonstrate cross‐country differences in both punishment and compensation as forms of third‐party intervention within the harm norm domain, highlighting cultural values as a key psychological mechanism underlying these differences. Practically, the use of ecologically valid judicial vignettes advances our understanding of how cultural orientations shape responses to justice‐related decisions.

## Ethics Statement

The research reported in this manuscript was approved by the ethics board of Hohai University.

## Conflicts of Interest

The authors declare no conflicts of interest.

## Supporting information


**Data S1:** pchj70061‐sup‐0001‐Supinfo.docx.

## Data Availability

The data that support the findings of this study are available from the corresponding author upon reasonable request.
