# Supplementary material for: Cultural Individualism–Collectivism and Third‐Party Punishment and Compensation
Source: Psych J. 2025 Oct 22;15(1):e70061. doi: 10.1002/pchj.70061 (PMC12897574; doi:10.1002/pchj.70061)
Supplement: Supplementary file 1 — Data S1: pchj70061‐sup‐0001‐Supinfo.docx. [file PCHJ-15-e70061-s001.docx]

**Supporting Information:**

**Cultural Individualism–Collectivism and Third-Party Punishment and Compensation**

**Content**

[S1: Experimental Materials 2](#_Toc2457)

[S2: Additional Analysis 5](#_Toc20939)

[S3: Mediation Analyses 7](#_Toc4327)

# S1: Experimental Materials

**A: Measurement of Subjective SES**

Please look at the ladder image with 10 steps on the right side. Imagine the steps on the ladder represent people’s social status in society:

-
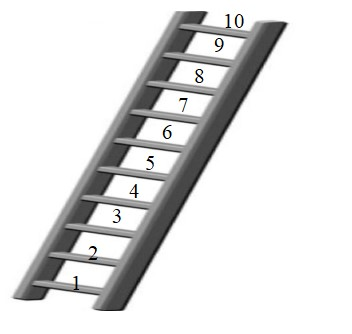
Those at the top step of the ladder are the ones with the highest income, highest education level, and the best and most prestigious jobs.
- Those at the bottom step of the ladder are the ones with the lowest income, lowest education level, and the least desirable and least respected jobs, or even no jobs.

Please fill in the number of the step that best represents your current position on this ladder. (Note: A higher number indicates a higher social status.)

**B: Judicial Scenarios**

In this study, we designed four judicial scenarios: Traffic Crime, Poisoning, Physical Assault, and Robbery. Each scenario included two outcome conditions: one with severe consequences and one with minor consequences. The specific scenarios are described as follows: (Names in parentheses correspond to those used in the Chinese version of the materials.)

**Scenario 1: Traffic Crime**

***Condition 1: Severe Consequences***

John [王某某], feeling frustrated by work difficulties and financial hardships, developed a desire to retaliate against society. On a morning in 2023, while driving through an intersection, he decided to express his resentment by hitting someone with his car. He accelerated and intentionally struck an innocent pedestrian, Mike [李某某], causing severe injuries to Mike’s leg and lower back.

***Condition 2: Mild Consequences***

John [王某某], feeling frustrated by work difficulties and financial hardships, developed a desire to retaliate against society. On a morning in 2023, while driving through an intersection, he decided to express his resentment by hitting someone with his car. He accelerated and intentionally struck an innocent pedestrian, Mike [李某某], causing minor injuries to Mike’s leg (a slight scratch).

**Scenario 2: Poisoning**

***Condition 1: Severe Consequences***

Alex [张同学] and Jordan [赵同学] are university roommates. Jordan is an excellent student with strong academic performance, while Alex struggles academically and lacks discipline. Out of jealousy, Alex decides to harm Jordan. One evening, after Jordan leaves the dorm for a while, Alex, left alone, seizes the opportunity to pour toxic chemicals obtained from the laboratory into Jordan's drink. Upon returning to the dorm and consuming the drink, Jordan suffers severe and irreversible physical damage.

***Condition 2: Mild Consequences***

Alex [张同学] and Jordan [赵同学] are university roommates. Jordan is an excellent student with strong academic performance, while Alex struggles academically and lacks discipline. Out of jealousy, Alex decides to harm Jordan. One evening, after Jordan leaves the dorm for a while, Alex, left alone, seizes the opportunity to pour toxic chemicals obtained from the laboratory into Jordan's drink. Upon returning to the dorm and consuming the drink, Jordan experiences discomfort. However, after a medical examination at the hospital, it is determined that there are no adverse consequences.

**Scenario 3: Physical Assault**

***Condition 1: Severe Consequences***

One day, Casey [周某] went out alone to have a meal. While preparing to leave, someone at the neighboring table, Morgan [赵某], approached and asked to exchange contact information. Casey politely declined Morgan’s request, but Morgan became enraged, resorting to verbal insults and physical assault. Casey sustained severe injuries and was sent to the hospital for treatment.

***Condition 2: Mild Consequences***

One day, Casey [周某] went out alone to have a meal. While preparing to leave, someone at the neighboring table, Morgan [赵某], approached and asked to exchange contact information. Casey politely declined Morgan’s request, but Morgan became enraged, resorting to verbal insults and physical assault. Fortunately, people nearby intervened in time, and Casey sustained only minor injuries.

**Scenario 4: Robbery**

***Condition 1: Severe Consequences***

Mark [郑某某], a 39-year-old unemployed individual, often wandered around a nearby park. One evening in 2022, as usual, Mark was strolling through the park when he noticed Mary [孙某某], who was walking alone. After scanning the surroundings and finding no one nearby, Mark followed Mary. In a secluded spot, Mark approached and attempted to mug Mary of her belongings and tried to assault her. During Mary’s resistance, Mark inflicted severe injuries on her.

***Condition 2: Mild Consequences***

Mark, a 39-year-old unemployed individual, often wandered around a nearby park. One evening in 2022, as usual, Mark was strolling through the park when he noticed Mary, who was walking alone. After scanning the surroundings and finding no one nearby, Mark followed Mary. In a secluded spot, Mark attempted to mug Mary of her belongings. Fortunately, an unrelated passerby happened to arrive at the scene, and Mark was unable to succeed in his attempt.

**C: Measurement of Third-Party Punishment and Compensation**

***Third-party Punishment***

Assume there is a grassroots organization seeking to achieve justice by crowdfunding to hire a renowned lawyer to increase the perpetrator’s punishment. As an ordinary citizen, if your daily income is 100 units, how many units would you be willing to contribute to support this cause?___ units.

***Third-party Compensation***

Assume there is a grassroots organization seeking to achieve justice by crowdfunding to hire a renowned lawyer to increase compensation for the victim. As an ordinary citizen, if your daily income is 100 units, how many units would you be willing to contribute to support this cause?___ units.

# S2: Additional Analysis

**A: Internal Consistency Across Scenarios**

To assess the consistency of participant responses across the four judicial scenarios, we calculated Cronbach’s alpha separately for punishment and compensation measures within each country. The results indicated excellent internal consistency for both outcomes (Chinese participants: *α*__punishment amount_ = 0.919, *α*__compensation amount_ = 0.883; American participants: *α*__punishment amount_ = 0.919, *α*__compensation amount_ = 0.883). Based on these high reliability values, we averaged responses across the scenarios to generate composite scores for each dependent variable per participant.

**B: Analysis of Residual Normality**

As the residuals of the raw data for both third-party compensation (see Panel A) and third-party punishment (see Panel B) deviated from normality, a Box-Cox transformation was applied to the data.

| *Panel A Compensation amount*  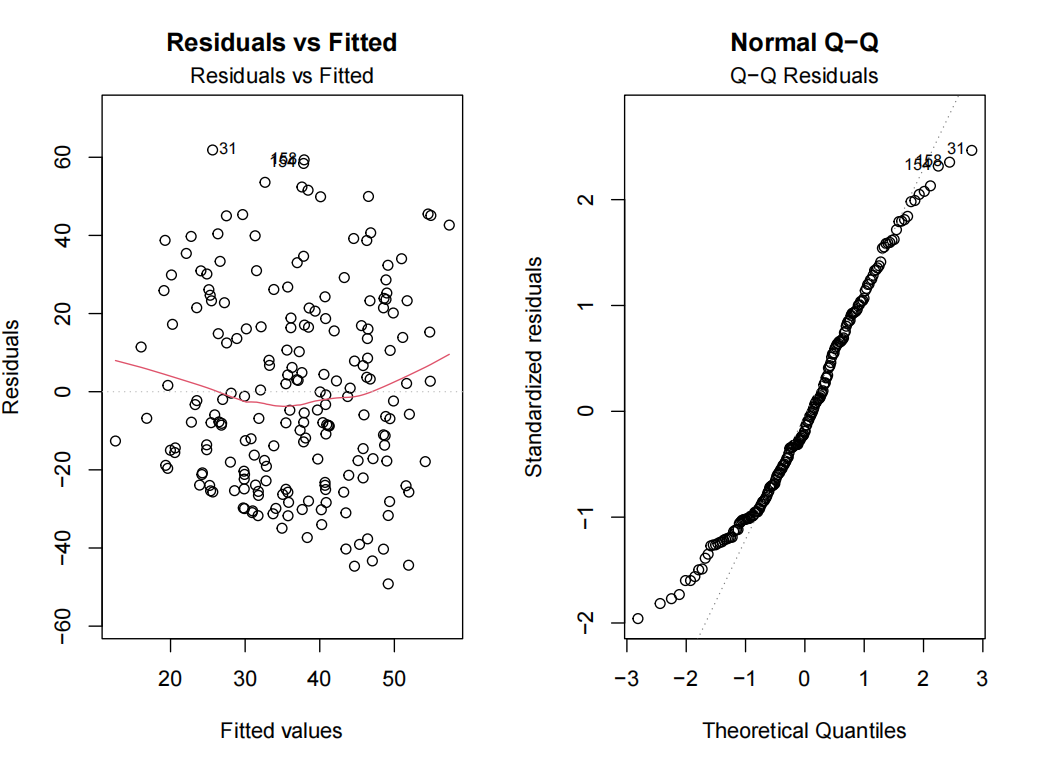 |
| --- |
| *Panel B Punishment amount*  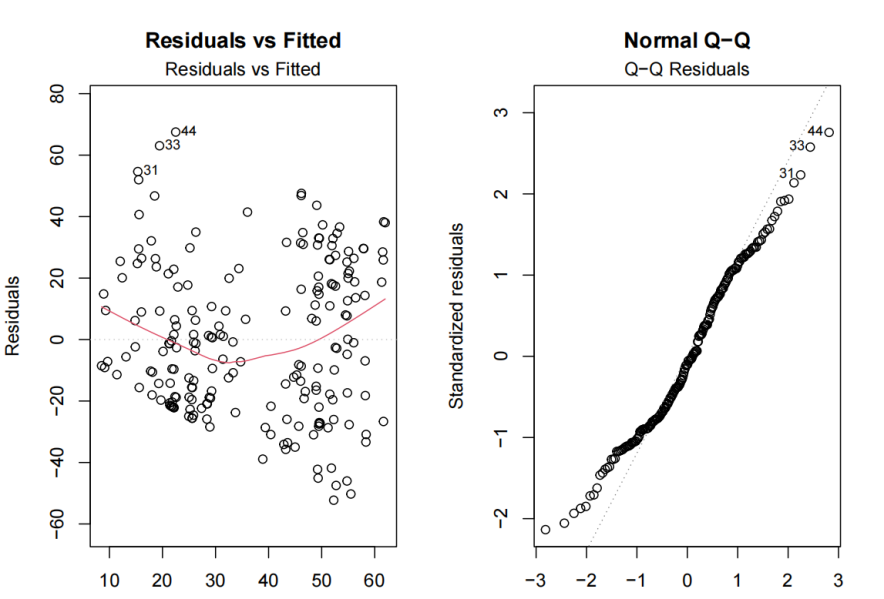 |

**C: Test of Cross-Country Differences in Perceived Consequence Severity**

To examine whether participants from individualism versus collectivism countries differed in their perceptions of consequence severity, we conducted a linear mixed-effects model with nation (USA vs. CN) and consequence severity (mild vs. severe) as fixed effects. Gender, age, and subjective SES were included as control variables. Results showed that the interaction between country and consequence severity was not significant (χ² = 1.69, *df* = 1, *p* = .196). Further analyses revealed that there were no significant differences in perceived severity between Chinese participants (*M*__mild_ = 5.39, *SE*__mild_ = 0.27; *M*__severe_ = 8.32, *SE*__severe_ = 0.27) and American participants (*M*__mild_ = 4.74, *SE*__mild_ = 0.28; *M*__severe_ = 8.34, *SE*__severe_ = 0.27) in either the mild or severe condition (*ps* > .10).

# S3: Mediation Analyses

**Table 1. Effect of Country on Third-Party Punishment via Individualism–Collectivism**

| Effect Type | Effect (b) | Proportion (%) | 95% CI |
| --- | --- | --- | --- |
| Direct Effect | −2.67 | 75 | [−3.63, −1.67] |
| Indirect Effect | −0.91 | 25 | [−1.50, −0.50] |
| Total Effect | −3.58 | 100 | [−4.60, −2.56] |

***Note:*** China coded as 0, USA coded as 1.

**Table 2. Effect of Country on Third-Party Compensation via Individualism-Collectivism**

| Effect Type | Effect (b) | Proportion (%) | 95% CI |
| --- | --- | --- | --- |
| Direct Effect | −1.06 | 48 | [−2.29, −0.08] |
| Indirect Effect | −1.17 | 52 | [−1.71, −0.54] |
| Total Effect | −2.23 | 100 | [−3.41, −1.09] |
